# From *in vivo* to *in vitro*: Major metabolic alterations take place in hepatocytes during and following isolation

**DOI:** 10.1371/journal.pone.0190366

**Published:** 2017-12-28

**Authors:** Shamir Cassim, Valérie-Ann Raymond, Pascal Lapierre, Marc Bilodeau

**Affiliations:** 1 Laboratoire d’hépatologie cellulaire, Centre de recherche du Centre hospitalier de l’Université de Montréal (CRCHUM), Montréal, Québec, Canada; 2 Département de médecine, Université de Montréal, Montréal, Québec, Canada; Universita degli Studi Di Cagliari, ITALY

## Abstract

The liver plays a key role in maintaining physiological homeostasis and hepatocytes are largely responsible for this. The use of isolated primary hepatocytes has become an essential tool for the study of nutrient physiology, xenobiotic metabolism and several liver pathologies. Since hepatocytes are removed from their normal environment, the isolation procedure and *in vitro* culture of primary hepatocytes is partially known to induce undesired metabolic changes. We aimed to perform a thorough metabolic profiling of primary cells before, during and after isolation using state-of-the-art techniques. Extensive metabolite measurements using HPLC were performed *in situ* in the liver, during hepatocyte isolation using the two-step collagenase perfusion method and during *in vitro* cell culture for up to 48 hours. Assessment of mitochondrial respiratory capacity and ATP-linked respiration of isolated primary hepatocytes was performed using extracellular flux analysis. Primary hepatocytes displayed a drastic decrease in antioxidative-related metabolites (NADPH, NADP, GSH and GSSG) during the isolation procedure when compared to the *in situ* liver (*P*<0.001). Parallel assessment of citric acid cycle activity showed a significant decrease of up to 95% in Acetyl-CoA, Isocitrate/Citrate ratio, Succinate, Fumarate and Malate in comparison to the *in situ* liver (*P*<0.001). While the levels of several cellular energetic metabolites such as Adenosine, AMP, ADP and ATP were found to be progressively reduced during the isolation procedure and cell culture (*P*<0.001), higher ATP/ADP ratio and energy charge level were observed when primary cells were cultured *in vitro* compared to the *in situ* liver (*P*<0.05). In addition, a significant decrease in the respiratory capacity occurred after 24 hours in culture. Interestingly, this was not associated with a significant modification of ATP-linked respiration. In conclusion, major metabolic alterations occur immediately after hepatocytes are removed from the liver. These changes persist or increase during *in vitro* culture. These observations need to be taken into account when using primary hepatocytes for the study of metabolism or liver physiopathology.

## Introduction

The liver is central to the homeostatic maintenance of physiological metabolism. Among its many functions, it is involved in the metabolism of carbohydrates with gluconeogenesis, glycogenolysis and glycogenesis and in the metabolism of lipids with the synthesis and degradation of triglycerides, of cholesterol into bile acids as well as in lipoprotein synthesis. It also ensures the synthesis of a large fraction of plasma proteins such as albumin and apolipoproteins. Hepatocytes are the cells chiefly responsible for these functions and they are known to constitute approximately 70% of all cells present in the normal liver [1]. Hepatocytes have an extremely active intrinsic metabolic activity that can be modulated to adapt to physiological needs [2].

However much remains to be understood about the physiology and metabolism of hepatocytes. Therefore, several experimental models for the study of hepatocyte physiology, pharmacokinetics of drugs and pathogenesis of liver diseases have been developed and many of those rely on the use of isolated primary hepatocytes [3–5]. These experimental models, used in both fundamental research and pharmacological studies, were developed based on the assumption that isolated hepatocytes shared similar metabolic profiles as the ones of hepatocytes *in situ* [6]. However, the original descriptions of theses similarities were made decades ago based on data acquired using tools and techniques available at the time [7–10].

Several studies that use primary hepatocytes use novel and sensitive techniques such as extracellular flux analysis to study the metabolism of isolated hepatocytes and conclude or propose hypotheses based on these results without knowing if the state of these primary hepatocytes reflect the state of hepatocytes *in vivo* [11, 12]. Therefore, a thorough characterization of the effect of isolation and *in vitro* culture on primary hepatocytes using the latest techniques is of the outmost importance if we are to continue to use isolated hepatocytes as models of hepatocytes *in vivo*.

Herein, we found that major metabolic alterations occur during both hepatocyte isolation and *in vitro* culture compared to liver cells *in situ*. These modifications include decreased antioxidative metabolite levels, decreased citric acid cycle activity, progressive reduction in cellular energetic metabolites and significant alteration in respiratory capacity. This metabolic plasticity of hepatocytes according to their environment needs to be taken in consideration in future *in vitro* studies using these cells as a substitute to *in vivo* liver studies.

## Materials and methods

### Animals and reagents

Male C57BL/6 mice (20g) were bought from Charles River (Saint-Constant, Qc, Canada) and fed *ad libidum* with normal chow. Animals were monitored daily for their appearance, state of hydration, behavior and clinical signs. Animals were sacrificed by exsanguination under anesthesia (induction with inhaled 4% Isoflurane and maintenance with inhaled 2% Isoflurane). All procedures were performed in accordance with the Canadian Council on animal care and approved by the *Comité institutionnel de protection animale (CIPA) du CHUM* (Canada). Dulbecco’s Modified Eagle Medium (DMEM), Leibovitz’s L-15 medium, Fetal Bovine Serum (FBS), Penicillin/Streptomycin were from Life technologies (Burlington, ON, Canada). Type IV Collagenase was from Wortington-Biochemicals inc. (Lakewood, NJ). Unless stated otherwise, all other products and chemicals were from Sigma-Aldrich (St-Louis, MO).

### Hepatocyte isolation and culture conditions

Hepatocytes were isolated from adult male C57BL/6 mice using the two-step collagenase perfusion method as previously described [13]. Briefly, under anesthesia, the peritoneal cavity was opened, and the liver was perfused *in situ* via the portal vein for 4 minutes at 37°C with Calcium-Magnesium(CM)-free HEPES buffer and for 7 minutes with CM-free HEPES buffer containing Type IV collagenase (35mg/100ml) and CaCl_2_ [10 mM]. Cells were used only if cell viability was above 80% as assessed by trypan blue exclusion. After three centrifugations (44g for 2min) in Leibovitz’s L-15 washing media supplemented with 0,2% bovine albumin, cells were seeded onto plastic petri dishes (26000cells/cm^2^). Cells were cultured in high glucose (25mM) DMEM supplemented with 10% FBS. All culture media contained penicillin (100units/ml) and streptomycin (100μg/ml). After cell attachment for 2 hours, the medium was replaced by fresh medium supplemented with 10% FBS. Primary cultures were kept in 5% CO_2_ atmosphere at 37°C for the indicated times: 0, 2, 4, 6, 24 and 48 hours following attachment.

### HPLC analysis

Metabolites were assessed using HPLC (Agilent 1200 HPLC system, Agilent technologies Canada inc., Mississauga, ON, Canada) by the Metabolomics Core Facility of CRCHUM (Canada). Metabolic measurements were performed before, during and after the isolation procedure: 1) *In situ* liver; 2) following HEPES perfusion; 3) after collagenase perfusion; 4) following washes in Leibovitz’s L-15 and 5) during *in vitro* culture between 0 and 48 hours after cell attachment. For *ex vivo* sample preparations, at each indicated steps and time points, liver samples or hepatocyte cell culture dishes (after removing culture medium) were snap frozen in liquid nitrogen and kept at -80°C until HPLC analyses. Glutamate levels were not measured for cells in culture due to the inclusion of L-glutamine in the culture medium. To compare metabolite quantification between hepatocytes in liver tissue and hepatocytes in cell culture, samples were normalized based on protein content using the Bradford method [14]. Nucleotide content is expressed in picomole per mg of proteins and the Energy Charge (EC) was calculated using this formula:

([ATP]+½[ADP])/([ATP]+[ADP]+[AMP]).

### Seahorse XF24 extracellular flux analysis

Cells (26000/cm^2^) were seeded on the XF24 microplate in high glucose DMEM supplemented with 10% FBS for the indicated times. According to manufacturer’s recommended protocol for Seahorse XF24 Extracellular Flux Analyzer, cell medium was replaced by conditional medium (culture medium without FBS and sodium bicarbonate) and incubated without CO_2_ for one hour before completion of sensor cartridge calibration. Oxygen consumption rate (OCR) was measured in a Seahorse XF24 Flux Analyzer (Agilent technologies Canada Inc., Mississauga, ON, Canada). Measurements were performed after injection of two compounds affecting bioenergetics: 10 mM glucose and 2 μM oligomycin. OCR was measured upon injection of glucose and ATP-linked respiration was calculated by measuring the decrease in OCR upon injection of oligomycin [15–17]. The measure of respiratory function could not be carried out reliably for the early steps of isolation (*In situ*, HEPES and Collagenase conditions) since glucose and oligomycin used in these assays did not adequately diffuse in hepatocytes maintained in tissue state (data not shown). Upon completion of the Seahorse XF24 Flux Analysis, cells were lysed to calculate the protein concentration. Results were normalized based on the total amount of proteins for each well.

### Statistical analysis

All data represent the values of at least three independent experiments from groups of 3 animals. Data are expressed as means ± standard error (SEM) and were analyzed with GraphPad Prism7 software (GraphPad Software, CA). Differences between groups were analyzed using the analysis of variance (ANOVA) test with Tukey or Dunnet post-test and student *t*-test. A *P* value below 0.05 was considered significant (* = P<0.05, ** = P<0.01, *** = P<0.001). All statistical tests were two sided.

## Results

### Primary hepatocytes show decresed levels of antioxidative stress-related metabolites following isolation and in vitro culture

**Fig 1:**
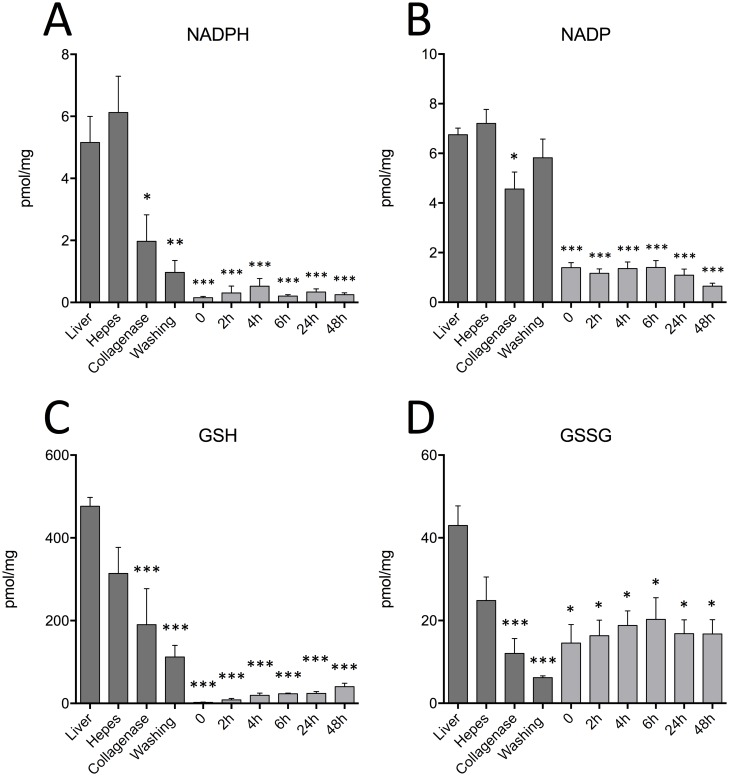
Decrease in antioxidative stress-related metabolites during the isolation procedure and cell culture. Evaluation of total intracellular: A) NADPH, B) NADP, C) GSH and D) GSSG levels during the hepatocyte isolation procedure from *in situ* to the washing step in L-15 media and during cell culture over a period of up to 48 hours. Values are ±SEM of 3 independent experiments. Asterisks indicate significance when compared to the *in situ* liver (*P<0.05, **P<0.01, ***P<0.001).

Primary hepatocytes displayed an important change in their antioxidative stress-related metabolites as shown by NADPH and NADP levels of isolated cells compared to *in situ* liver (*P*<0.001) (Fig 1A and 1B). This decrease of up to 95% was observed during the earliest steps of the isolation procedure, collagenase perfusion, where both NADPH and NADP contents are reduced significantly (*P*<0.05) but also during cell culture from 0 to 48 hours where levels are further reduced (*P*<0.001). Interestingly, NADPH/NADP ratio also showed a significant decrease in all the different conditions in comparison to the *in situ* liver (*P*<0.01) (S1B Fig). The levels of reduced and oxidized forms of glutathione, GSH and GSSG, were also affected by isolation and culture. Levels of GSH and GSSG were reduced by up to 85% during collagenase perfusion and washing steps (*P*<0.001) but also during *in vitro* cell culture, (*P*<0.001 and *P*<0.05, respectively) (Fig 1C and 1D). Moreover, primary cultured hepatocytes showed a reduction of 79% of the GSH/GSSG ratio compared to the *in situ* liver (*P*<0.05) whereas the HEPES, collagenase and washing steps did not lead to any significant modification of this ratio (S1C Fig). In parallel, glutamate levels were also measured during the isolation procedure since it is one of the main precursor of glutathione. A lower content of glutamate was observed after the collagenase (*P*<0.05) and washing step (*P*<0.01) when compared to the *in situ* liver (S1A Fig).

### Levels of citric acid cycle metabolites are significanlty altered during isolation and culture

**Fig 2:**
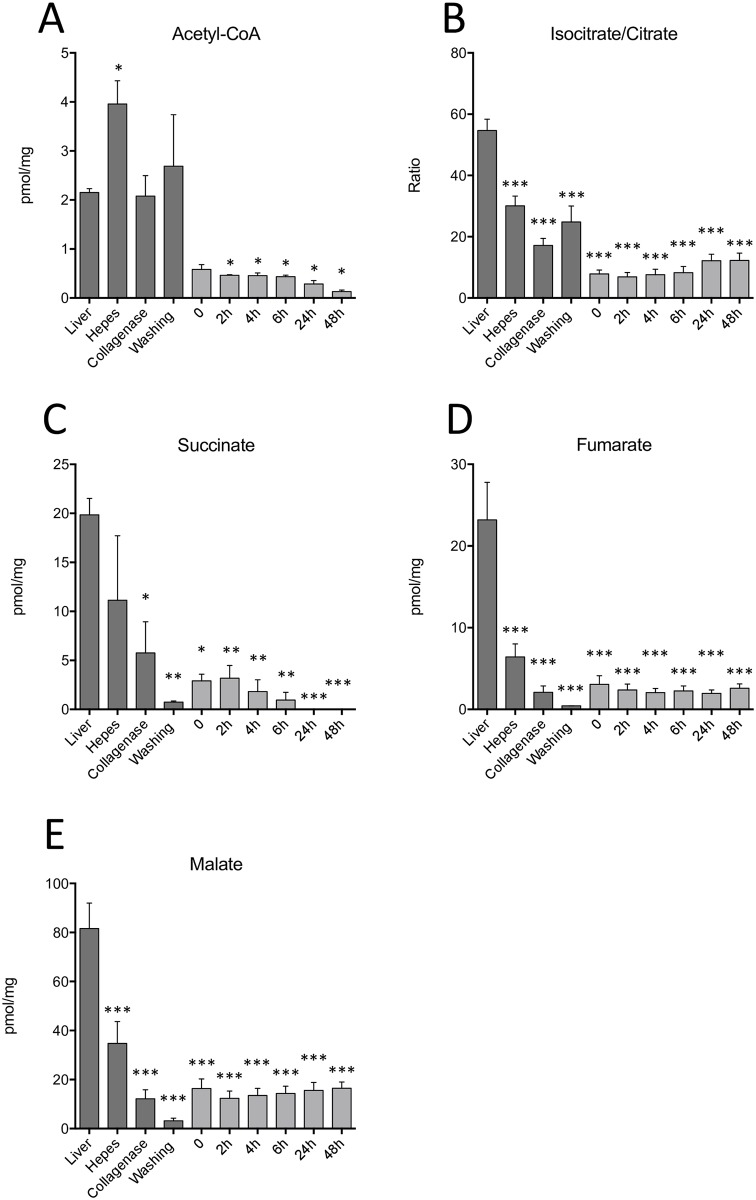
Reduction in TCA metabolites during the isolation procedure and cell culture. Assessment of intracellular: A) Acetyl-CoA, B) Isocitrate/Citrate ratio, C) Succinate, D) Fumarate and E) Malate during the hepatocyte isolation procedure and cell culture for a period of up to 48 hours. Values are ±SEM of 3 independent experiments. Asterisks indicate significance when compared to the *in situ* liver (*P<0.05, **P<0.01, ***P<0.001).

The citric acid cycle or Krebs cycle is central to the energy metabolism of hepatocytes: it is possible that the alteration in the microenvironment of hepatocytes induced by cell isolation and culture result in changes in this crucial pathway. Acetyl-CoA levels were indeed decreased by 94% when primary hepatocytes were cultured *in vitro* as early as 2 hours after cell attachment (*P*<0.05) (Fig 2A). The isocitrate/citrate ratio was decreased by up to 68% during the isolation procedure and remained low throughout the cell culture (*P*<0.001) (Fig 2B). Succinate displayed reduced levels not only during the isolation process (collagenase (*P*<0.05) and washing step (P<0.01)) but also during *in vitro* culture (*P*<0.05 and *P*<0.001) (Fig 2C). When Fumarate and Malate quantifications were performed, the same metabolic profile was observed: a strong reduction ranging from 57% to 98% of their abundance in the early steps of isolation in both HEPES (*P*<0.001), collagenase (*P*<0.001) and during washes (*P*<0.001) and low levels of both metabolites throughtout *in vitro* culture (*P*<0.001) (Fig 2D and 2E). Although no significant differences were found in NADH content (S2A Fig), NAD levels were strongly reduced during collagenase perfusion (*P*<0.001) and cell culture, by up to 60% and 93%, respectively (*P*<0.001) (S2B Fig). This led to significant increases in NADH/NAD ratio during these steps (S2C Fig).

### Primary hepatocyte isolation and cell culture alters the level of cellular energetic metabolites

**Fig 3:**
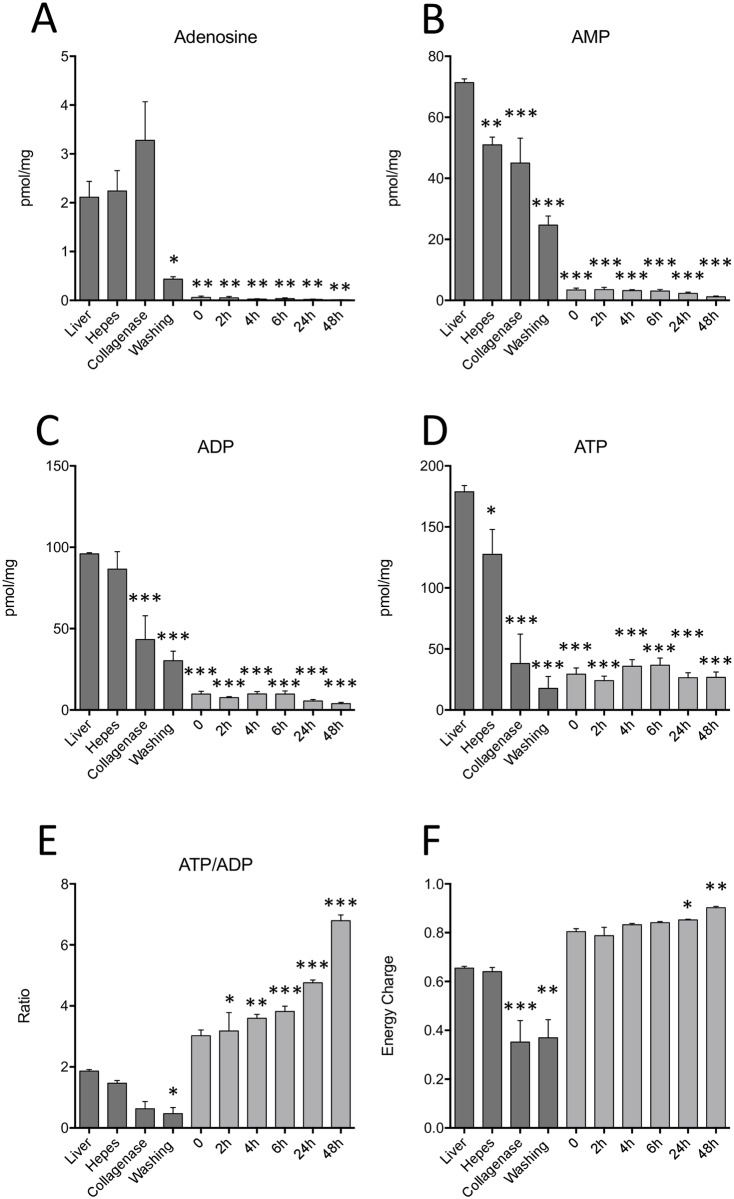
Reduced levels of cellular energetic metabolites during isolation and cell culture. Quantification of total intracellular hepatic A) Adenosine, B) AMP, C) ADP, D) ATP and E) ATP/ADP ratio during the isolation procedure and cell culture for a period of up to 48 hours. F) Calculated energy charge values during isolation and *in vitro* culture. Values are ±SEM of 3 independent experiments. Asterisks indicate significance when compared to the *in situ* liver (*P<0.05, **P<0.01, ***P<0.001).

The above changes in levels of citric acid cycle intermediates suggest that cellular energetic metabolites could also be altered in isolated and cultured hepatocytes. To characterize this possible alteration, the levels of Adenosine were measured: a significant decrease ranging from 79% to 99% was observed from the last isolation steps but most significantly during cell culture (*P*<0.01) (Fig 3A). AMP, ADP and ATP levels were also measured and like Adenosine these three energetic metabolites showed a drastic reduction in the early stages of the isolation process such as the collagenase (*P*<0.001) and washing (*P*<0.001) steps but mostly during cell culture (*P*<0.001) (Fig 3B–3D). In addition, the ATP/ADP ratio and energy charge levels were at least ten times greater during *in vitro* culture in comparison to the *in situ* liver (Fig 3E and 3F).

### Cell culture impacts the mitochondrial respiratory capacity of primary hepatocytes

**Fig 4:**
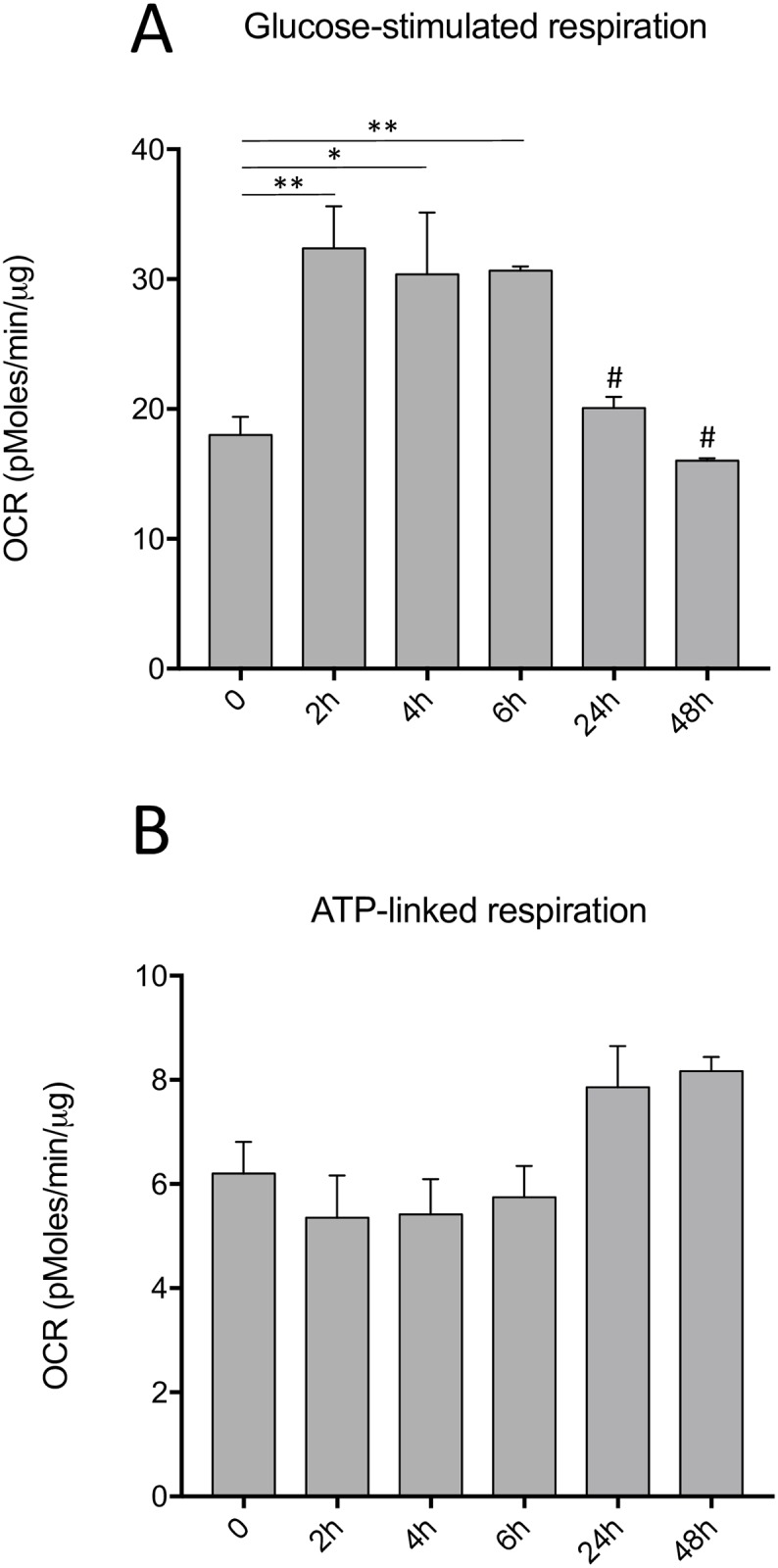
Primary hepatocyte cultures show a significant decrease in mitochondrial oxygen consumption rate but stable mitochondrial ATP production. Extracellular flux analysis was used to measure the mitochondrial function of primary hepatocytes cultured for 0 to 48 hours. A) Mitochondrial respiration (OCR levels) after glucose stimulation (10 mM). B) Assessment of ATP-linked respiration following the addition of oligomycin (2 μM). Values are ±SEM of 3 independent experiments (*P<0.05, **P<0.01*; # P*<0.05 when compared to primary hepatocytes cultured for 2,4 and 6 hours).

Glucose-stimulated respiration assesment of cultured hepatocytes showed that cells in culture for 2, 4 and 6 hours had a greater mitochondrial respiration rate than freshly seeded hepatocytes (0h) (respectively *P*<0.01, *P*<0.05 and *P*<0.01) (Fig 4A). Interestingly, hepatocytes maintained in culture over an extended period, such as 24 and 48 hours, showed a significant drop in mitochondrial respiratory capacity when compared to those cultured for 2, 4 and 6 hours (P<0.05) (Fig 4A). However, ATP-linked respiration, a measure of the ability of cells to generate ATP through mitochondrial respiration, did not show any significant alteration during cell culture (Fig 4B).

## Discussion

Primary liver cells have been the object of study from both a morphological and a functional aspect for several years. It is by the end of the 1970s that the use of primary hepatocytes in liver research became mainstream notably thanks to Bissell and Seglen who revolutionized both the procedure for their isolation and their *in vitro* culture [10, 18]. These technical innovations paved the way for novel *in vitro* studies such as those on the hepatic metabolism of drugs and hepatotoxicity [7]. Cultured hepatocytes were shown to maintain their crucial functions, such as respiration [8], lipid synthesis [9], albumin synthesis [10], and the ability to modulate their glycogen storage following insulin/glucagon stimulation [10]. Thus, isolated hepatocytes have been considered an excellent model to study liver metabolism and liver diseases *ex vivo*. However, considering the metabolic plasticity of hepatocytes *in vivo*, it is conceivable that isolated hepatocytes could modulate their metabolic activity based on their surrounding environment and therefore, differ metabolically from hepatocytes *in situ*.

Some metabolic alterations of primary hepatocytes have been reported, including a decrease in mitochondrial respiration according to culture conditions [8], ATP generation [10] or a lower level of total glutathione [19]. Our study aimed at defining the metabolic profile of hepatocytes from *in situ* to *in vitro* during their isolation using modern techniques with the aims of evaluating the extent of the metabolic changes that occurrs at each steps of the isolation procedure and during *in vitro* culture.

In this study, we chose *in vitro* culture conditions based on what is frequently used in studies with primary hepatocytes. To limit the introduction of any bias from prolonged *in vitro* culture, hepatocytes were cultured for a maximum of 48 hours without addition of glucocorticoids. Dexamethasone has been used in cell culture medium to improve and prolong the differentiation status of serum-containing cell cultures [20–23]. Dexamethasone can be useful for the long-term preservation of hepatocyte-specific functions, polyglonal hepatocyte morphology, as well as the structural integrity of cytoplasmic membranes, especially bile canaliculi-like structures [22, 23]. However, its use would have been a confounding factor for the analysis of metabolic changes occurring in hepatocytes kept in culture when compared to those found *in situ*. Primary hepatocytes were also directly plated on plastic petri dishes since this is the most frequently used method in studies with short-term *in vitro* culture of primary hepaticytes. However, matrix overlays (layers of gelled extracellular matrix proteins with collagen I and matrigel), 3-dimensional culture systems or improved air-liquid interface have been shown to dramatically influence the morphological development, cellular and metabolic functions, and survival of hepatocytes in culture [24–26].

The results herein show that there is an important alteration in antioxidative metabolites levels that occurs during and following the isolation process. A significant decrease in NADPH, NADP, GSH and GSSG levels were observed at each step of the isolation procedure compared to the *in situ* liver. Since oxidative stress can occur during the isolation step, especially during the collagenase step perfusion, it could affect the antioxidative status of cells [27]. These metabolic changes occurred early in the isolation process and were sustained during cell culture. Previously, minor modifications in the antioxydative status of isolated primary hepatocytes have been reported but modifications to NADPH and NADP were not assesed and levels of GSH and GGSG were found to fluctuate but remain within normal range during culture based on their measurement using an enzymatic assay [28].

In agreement with the decrease microsomal activity of cytochrome P450 enzymes previously described during isolation [29], our results show significantly reduced levels of NADPH, a molecule essential for the reducing activity of cytochrome P450 and the biodegradation of xenobiotics [30, 31]. Interrestingly, previous studies had shown a significant decrease in catalase activity, an enzyme that protects cells from reactive oxygen species (ROS), in freshly isolated rat hepatocytes in comparison to liver homogenates [27, 32].

The citric acid or tricarboxylic acid (TCA) cycle, is a major metabolic pathway; its primary function is to oxidize acetyl groups to recover energy through electron transfer, subsequently allowing the generation of ATP by the mitochondrial respiratory chain [33]. Moreover, the TCA cycle occurs at the junction of several other major metabolic pathways such as gluconeogenesis and the oxidation of fatty acids [33]. Surprisingly, decreased levels of the citric acid cycle intermediates acetyl-CoA, isocitrate/Citrate, succinate, fumarate and malate were observed during the isolation and *in vitro* culture. These metabolic alterations during the various steps of isolation could therefore have a significant impact on primary hepatocyte functionallity and especially, their metabolic phenotype. Therefore, attention should be paid to studies that previously focused on TCA activity in isolated primary hepatocytes because it may not perfectly reflect the state of hepatocytes *in situ* [34, 35].

ATP is a crucial energy molecule and while it is important for cellular function in general, it is also important for the homeostatic properties specific to liver cells [36]. Although a constant decrease in AMP, ADP and ATP was observed during the different steps, a significant increase in the ATP/ADP ratio and energy charge was also noted during cell culture. While this energy charge ratio is based on small absolute values and could be influenced by small fluctuations in absolute levels of AMP, ADP or ATP, it is stable over time and significantly higher than the energy charge of liver cells *in situ*. These observations could be explained by the fact that hepatocytes maintained in culture for 24 to 48 hours gradually loose their intrinsic characteristics of intense metabolic activity thereby decreasing their respiratory rate or, simply by the fact that an increased mitochondrial respiratory capacity possibly take place within these cells. However, an increased respiratory capacity would be contrary to previous observations whereby a reduction in oxygen uptake is observed in hepatocytes maintained in culture media [8]. In addition, reactivation of respiratory capacity would likely have a positive impact on ATP content as opposed to the decreased levels we oberved.

The analysis of respiratory function by extracellular flux analysis allowed us to detect a significant increase in oxygen consumption rate by the hepatocytes maintained in culture for 2 to 6 hours, compared to baseline (t = 0). However, hepatocytes cultured for 24 to 48 hours showed a significant decrease in their respiratory capacity in comparison to the earlier timepoints. On the other hand, their ATP-linked respiration did not significantly vary during isolation or cell culture. Therefore, the posibility that the increased ATP/ADP ratio and energy charge is the consequence of an increased respiratory capacity is not confrimed by our observations since hepatocytes significantly decreased their oxygen consumption rate at 24 and 48 hours, this even though ATP-linked respiration levels remained stable. Thus, we believe that hepatocytes that remain in culture for longer periods of time, will see their intense metabolic activity being gradually reduced to a state reminiscent of a quiescent cell.

Interestingly, studies on the dedifferentiation of primary hepatocytes in culture found early transcriptomic modifications in these cells as early as four hours after plating [26, 37, 38]. These include genes related to the TCA cycle, mitochondrial dysfunction and oxidative phosphorylation [37]. These transcriptomic modifications led to proteomic modifications 24 hours after plating and these modifications were driven by non-coding microRNAs (miRNAs) [37]. These studies reveal how hepatocyte can respond rapidly to changing conditions and studies that rely on primary hepatocyte should ensure, either through metabolic assays as described herein or assessment of dedifferentiation through transcriptomic or proteomic assays[26, 37, 38] that primary hepatocyte maintain their phenotype for the duration of their *in vitro* studies.

In conclusion, isolation of primary hepatocytes leads to a decrease in antioxidative-related metabolites, citric acid cycle intermediates and also several energetic metabolites. A greater ATP/ADP ratio and energy charge level and a decrease in the respiratory capacity are observed when these cells are cultured *in vitro* but ATP-linked respiration remains unchanged. Some of these metabolic alterations occur immediately after hepatocytes are removed from the liver and persist during *in vitro* culture under standard conditions. These metabolic changes will need to be taken into account in studies that use primary hepatocytes and assess metabolic parameters.

## Supporting information

S1 Fig Reduced levels of glutamate and NADPH/NADP and GSH/GSSG ratios during primary hepatocyte isolation and cell culture. **A)** Quantification of total intracellular hepatic glutamate during the isolation of primary hepatocytes. B) NADPH/NADP and C) GSH/GSSG ratios during isolation and culture for a period of up to 48 hours. Values are ±SEM of 3 independent experiments. Asterisks indicate significance when compared to the *in situ* liver (*P<0.05, **P<0.01).
(TIF)

S2 Fig Levels of NADH and NAD during isolation and cell culture. Quantification of total intracellular hepatic A) NADH, B) NAD and C) calculated NADH/NAD ratio during the isolation procedure and cell culture for a period of up to 48 hours. Values are ±SEM of 3 independent experiments. Asterisks indicate significance when compared to the *in situ* liver (**P<0.01, ***P<0.001).
(TIF)
