# Supplementary figures and images for: From in vivo to in vitro: Major metabolic alterations take place in hepatocytes during and following isolation
Source: PLoS One. 2017 Dec 28;12(12):e0190366. doi: 10.1371/journal.pone.0190366 (PMC5746264; doi:10.1371/journal.pone.0190366)

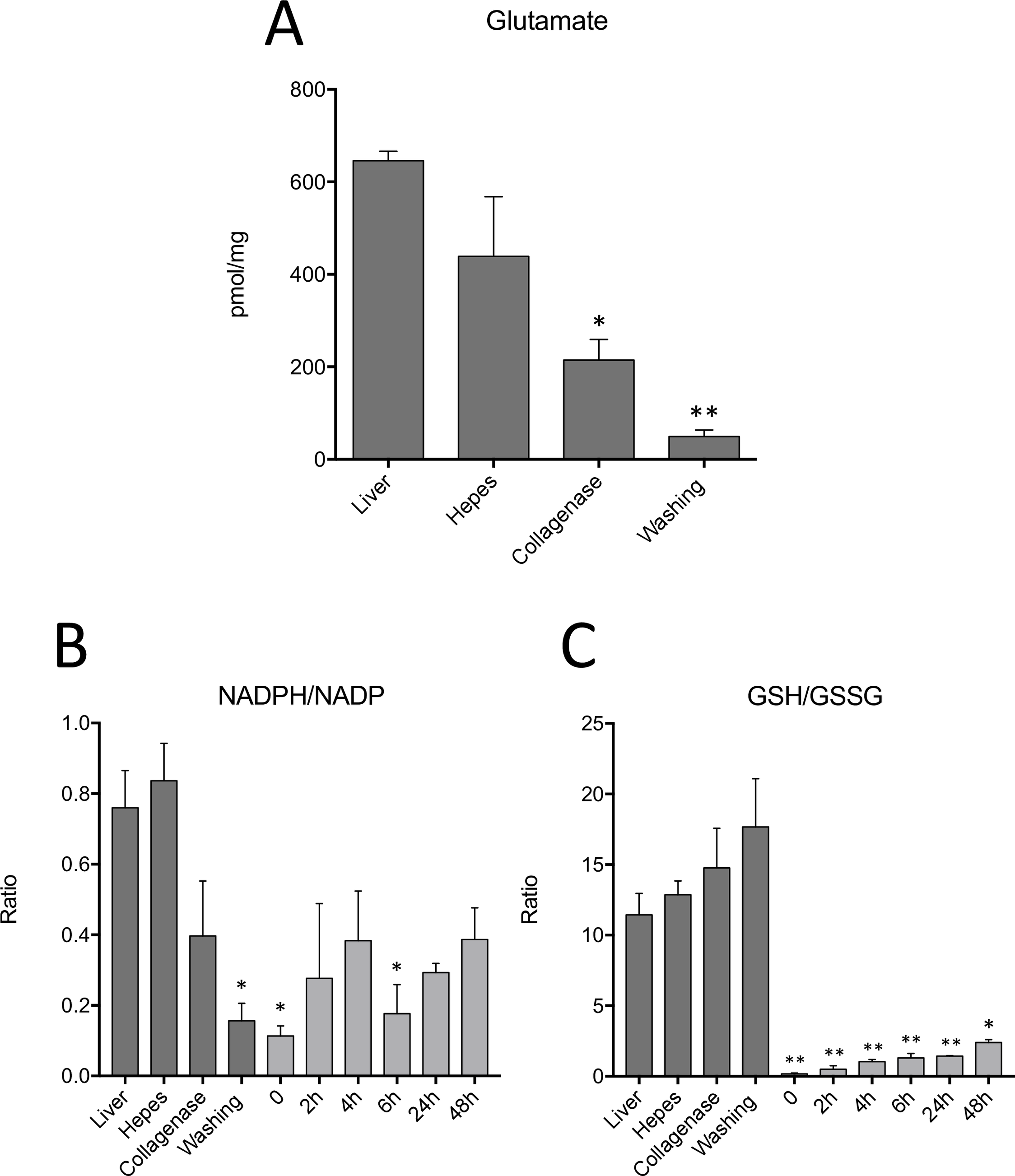

Supplement: S1 Fig — A) Quantification of total intracellular hepatic glutamate during the isolation of primary hepatocytes. B) NADPH/NADP and C) GSH/GSSG ratios during isolation and culture for a period of up to 48 hours. Values are ±SEM of 3 independent experiments. Asterisks indicate significance when compared to the in situ liver (*P<0.05, **P<0.01). (TIF) [file pone.0190366.s001.tif]

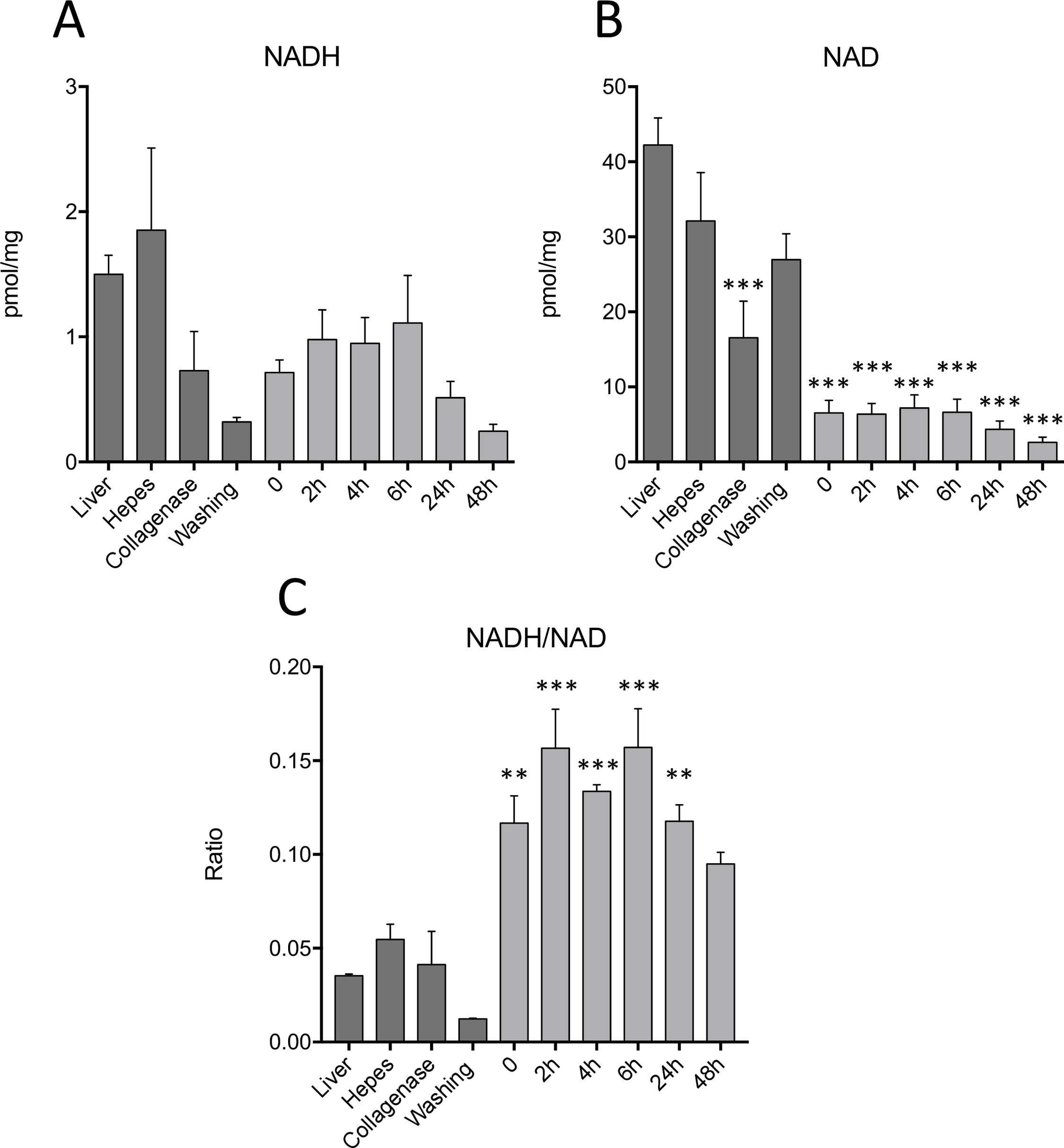

Supplement: S2 Fig — Quantification of total intracellular hepatic A) NADH, B) NAD and C) calculated NADH/NAD ratio during the isolation procedure and cell culture for a period of up to 48 hours. Values are ±SEM of 3 independent experiments. Asterisks indicate significance when compared to the in situ liver (**P<0.01, ***P<0.001). (TIF) [file pone.0190366.s002.tif]
